# Job competence gap of quantity surveying graduates of higher vocational colleges with a high dimensional measurement error model

**DOI:** 10.1371/journal.pone.0328617

**Published:** 2026-02-09

**Authors:** Dongmei Huangfu, Yun Fah Chang

**Affiliations:** 1 Department of Architectural Engineering Institute, Dianchi College, Kunming, Yunnan, China; 2 School of Accounting & Finance, Faculty of Business & Law, Taylor’s University, Subang Jaya, Selangor, Malaysia; University of Botswana, BOTSWANA

## Abstract

The primary aim of this study is to measure the competency gaps between industry expectations and current competencies of recent Chinese quantity surveying graduates from higher vocational colleges, using self-reported data. An innovative measure with a high-dimensional measurement error model was utilized to evaluate competency gaps. We used a quantitative technique with purposive sampling to collect data via a questionnaire survey of quantity surveying graduates with a higher vocational diploma who had worked in China for no more than three years. The findings indicate a competency gap in job matching between quantity surveying higher vocational college graduates’ performance and industry expectations. The personality competency gap is medium, whereas the knowledge, skills, and ethical gaps are minimal. The top three gaps are in business administration, personal image, and innovative insight. The study’s findings highlight the need for training programs focused on management and personality development for quantity surveying higher vocational college graduates. They also emphasize the importance of quantity surveying professionals acquiring interdisciplinary skills through educational programs that combine management with personality attributes such as personal image and innovative insight.

## Introduction

Quantity surveyors (QSs), known as “cost engineers” in China, serve as the bedrock for cost management, contractual agreements, and financial control in construction projects. Higher vocational colleges (HVCs) are a primary source for this workforce, offering three-year, practice-oriented programs designed to create job-ready technicians. However, a critical mismatch persists between HVC training and the industry’s rapid evolution toward digitalization, sustainability, and complex management. QS curricula often lag, overemphasizing theory at the expense of practical, integrated competencies. Consequently, graduates possess foundational skills but face an immediate competency gap, lacking the sophisticated management and professional skills demanded by modern firms.

Existing research has broadly acknowledged this industry-education misalignment. Previous studies have effectively mapped the evolving skill sets required for QS professionals [1], documented industry perceptions of graduate preparedness [2], and evaluated the efficacy of specific pedagogical tactics [3]. However, the methodologies employed to precisely quantify this multifaceted gap often present limitations. Some studies rely predominantly on qualitative approaches that limit generalizability [1], while others use surveys that may not fully capture the high-dimensional nature of competencies across different domains [2]. Consequently, there remains a lack of a robust, quantitative methodology capable of simultaneously evaluating the complex interplay between knowledge, skills, personality, and ethics, while accounting for the inherent measurement errors in self-reported survey data.

To address this methodological gap, this study introduces a novel analytical approach. We posit that a precise diagnosis requires a model that treats both industry expectations and graduate competencies as multidimensional constructs measured with error. Therefore, this research employs a high-dimensional measurement error model—specifically, the multidimensional unreplicated linear functional relationship model (MULFR) [4]—to quantify the competency gaps. This approach allows us to move beyond descriptive summaries and provide a statistically rigorous, high-dimensionality-specific similarity measure (the coefficient of determination) to evaluate the gaps.

The primary aim of this research is to investigate the gaps between industry expectations and the competencies of Chinese QS HVC graduates across multiple dimensions (knowledge, skills, personality, and ethics). By doing so, this study makes two key contributions: (a) it provides a precise, multidimensional diagnosis of the specific competency gaps, identifying priority areas for intervention (e.g., business administration, personal image); and (b) it demonstrates the application of an innovative statistical model (MULFR) for competency gap analysis, offering a methodological advancement that enhances the accuracy and reliability of findings in this field. The outcomes are poised to directly inform curriculum reform in HVCs and talent development strategies in the Chinese construction industry.

### Theoretical background and competency indicators’ framework

#### Changes in competency requirements for quantity surveyors.

Propelled by massive domestic and international projects, particularly under the Belt and Road Initiative (2013-present), China has emerged as a global infrastructure powerhouse over the past decade [5]. The resultant boom in the construction industry is clearly demonstrated by the accompanying charts tracking its growth from 2016 to 2021.

Quantity surveyors were initially limited to working for a project’s consultant, contractor, or client [6]. They are now employed as project managers, arbitrators, and other specialists in fields such as insurance, finance, manufacturing, taxation, and valuation [7]. In the future, they may need to master entirely new disciplines, including development appraisal, life cycle costing, sustainable construction management, supply chain management, quality assurance coordination [8], and facilities management [9,10]. Their engagement in these new sectors will require a diverse set of capabilities to meet the industry’s evolving demands.

The Ministry of Education of the People’s Republic of China now establishes training requirements based on teaching quality standards for QS majors at Chinese universities, as outlined in the document *Project Cost Teaching Requirements in Higher Vocational Schools (2019)*. The sector is rapidly changing, and graduate quantity surveyors must improve their abilities in order to move into various types of enterprises. Diversifying tasks would make graduates more employable [11,12]. As a result, it is critical to determine whether the training standards for QS majors at Chinese HVCs meet the industry’s talent requirements.

### Research related to quantity surveyors’ competency gap

Several studies have investigated different aspects of quantity surveyor competencies and job satisfaction, employing a variety of approaches to analyze current trends and identify gaps. Attakora-Amaniampong employed Structural Equation Modeling (SEM) to establish significant linkages between key competency groups in Ghanaian building and construction firms [13,14]. Alroomi et al. used SEM to evaluate methods for retaining cost-estimating competencies [15]. Dada and Jagboro utilized the Delphi survey technique to relate essential training standards in education, professional competence, and professional development [16], as well as to establish parameters for surveyor competency. Yogeshwaran et al. employed a hybrid method that included expert interviews, desk reviews, and a questionnaire survey, alongside Bloom’s Taxonomy, to assess the competency levels of graduate quantity surveyors [7]. Lian and Ling aimed to assess quantity surveyors’ job satisfaction and identify personal factors that influence job satisfaction using self-administered QS questionnaires [17]. Oke et al. examined the current and significant competency areas of quantity surveyors in Nigeria, using an Average Competency Indicator Score (MIS) to rank required and performance competencies, and employed gap analysis, quadrant analysis, and the Mann-Whitney U test to investigate the gap between required and exhibited competencies [18]. Arun et al. identified the various roles that quantity surveyors are currently expected to perform, as well as the skills and competencies needed for these roles [10]. Their mixed-methods approach, which comprised semi-structured interviews and a questionnaire survey, identified the top 10 competencies, including contract documentation, construction technology, and project management. Nattariga et al. used a literature survey, the Delphi method, and the Index of Item-Objective Congruence (IOC) to determine that the largest gaps were in delivery management, data usage, and data analytics, while the smallest gaps were in innovation management, cloud usage, and ICT functionalities, highlighting strengths in the industrial engineering degree program [19].

These studies help to better identify the competencies, job satisfaction, and opportunities for improvement in the QS profession. Table 1 summarizes the key findings and methodologies of selected competency-related studies on quantity surveyors.

**Table 1:** Key outcomes and method of QS competency gap related research.

Author	Key outcomes	Method
Attakora Amaniampong E.	Create favourable strong links between the critical competency groups for Ghanaian building construction enterprises	SEM
Alroomi, et al.	Evaluated the strategies for maintaining cost-estimating skills.	SEM
Dada & Jagboro	Link the fundamental standards of training in education, professional competence and professional development.	Delphi survey research approach
Yogeshwaran et al.	Determined the competency levels of graduate quantity surveyors	A hybrid method of expert interviews, desk reviews and a questionnaire survey. Bloom’s Taxonomy.
Lian & Ling	Personal characteristics influence their job satisfaction	Self-administered quantity surveyors questionnaire
Oke et al.	Examine the gap between required and exhibited competencies of Nigerian quantity surveyors。	Average competency indicator score. Mann–Whitney U test
Arun et al.	The 10 most important competencies: contract documentation, construction technology, contract administration, project management, dispute resolution, cost estimating, life cycle cost analysis, budgetary process, value management and cost planning.	Mixed approach comprising semi-structured interviews and a questionnaire survey, data with mean rank method.
Nattariga et al.	The greatest gaps: delivery management, data usage, and data analytics in the usage phase. The smallest gaps: innovation management, cloud usage, ICT add-on functionalities and technology in production.	Literature survey. Delphi method. Indicator of Competency Indicator Objective Congruence(IOC)

### Onion model and Person-Environment fit theory

The onion model is a conceptual framework that is widely applied in the disciplines of organizational behaviour, human resource development, and competency gap analysis. It employs a multifaceted approach to understanding complex phenomena, including competencies, organizational culture, and individual behaviours. This approach helps to break down these components into manageable layers, allowing for deeper examination and more effective solutions.

**Fig 1:**
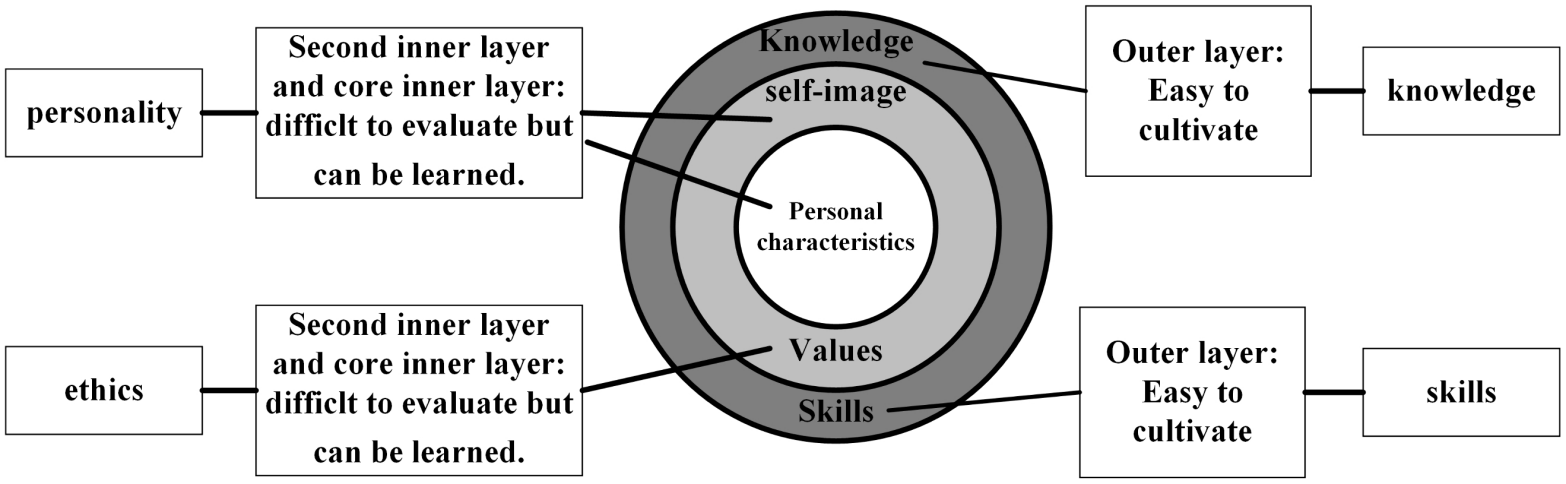
The onion model and the corresponding competency domains.

The onion model, introduced by American researcher Richard Boyatzis, posits that the outer layer of human qualities is easier to assess than the inner layer, which is more difficult. As in the onion model, we proceed from the outer layer of necessary knowledge and skills to the inner layer of underlying individual attributes [20]. Consequently, Fig 1 displays the competency elements for newly graduated quantity surveyors in China, which encompass four components: knowledge, skills, ethics, and personality.

The onion model has been applied in various fields, including but not limited to: (a) Competency gap analysis: In human resource development, the onion model is used to deconstruct the competencies required for various roles. This approach aids in identifying gaps between existing and desired competencies, thereby facilitating the design of more effective training and development programs [21]. (b) Educational frameworks: The onion model can assist educators in designing curricula that address not only surface-level knowledge and skills but also underlying attitudes and values. This comprehensive approach promotes deeper learning and personal development [20].

The onion model provides a robust foundation for analyzing complex constructs by deconstructing them into manageable layers. Its application across various disciplines demonstrates its adaptability and effectiveness in addressing deep-seated issues within organizations and individuals. By understanding and addressing each layer, practitioners can implement more comprehensive and sustainable improvements.

This study is grounded in Person-Environment (P-E) Fit Theory [22], which posits that the congruence (or lack thereof) between individual attributes and environmental characteristics leads to critical outcomes such as job performance, satisfaction, and retention. In the context of this research, the “Person” is operationalized as the multidimensional competencies possessed by QS graduates, while the “Environment” is represented by the multidimensional competency expectations of the construction industry. A misalignment between the graduate and the professional environment constitutes a “misfit,” which this study aims to measure precisely. The “Onion Model” serves as the conceptual map that defines and organizes these multidimensional attributes for both the person and the environment, structuring the constructs to be measured.

### Competency indicators

Competency is a set of skills, knowledge, and attributes that lead to outstanding performance in professional practice [23]. Personal characteristics, abilities, applied knowledge, and skills can all indicate competency. These competencies enable individuals to perform their stated professional duties [23]. Furthermore, competence encompasses knowledge, skills, abilities, and personality traits [24]. Balve and Ebert classified engineering students’ competencies into four categories: professional competencies (e.g., knowledge of information technology/IT), methodological competencies (problem-solving ability, analytical skills), social competencies (oral expression, communication skills), and personal competencies (adapting to changing circumstances, decision-making ability) [24,25]. Consequently, both industry and academia consider measuring competency within four major domains: knowledge, skills, ethics, and personality.

The selection of the four core domains—knowledge, skills, personality, and ethics—as the foundational framework for this study is grounded in both established educational psychology models and the specific competency standards recognized within the global quantity surveying profession. First, the framework is conceptually anchored in the widely adopted “Onion Model” [20], which posits that competencies range from outer, easily observable layers (knowledge, skills) to inner, more intrinsic attributes (ethics, personality). This layered approach provides a comprehensive structure for deconstructing complex professional competence. Second, and more critically, this four-domain structure directly aligns with the competency taxonomies consistently emphasized in quantity surveying literature and professional guidelines. For instance, studies have categorized QS competencies into similar groupings, such as professional/methodological/social/personal competencies [24,25], or have explicitly highlighted the intertwined importance of technical know-how (knowledge/skills), professional conduct (ethics), and personal attributes (personality) for career success [7,10,17,26]. By integrating the conceptual clarity of the Onion Model with the empirically derived competency expectations from the QS field, this framework ensures that the assessment is both theoretically robust and professionally relevant, covering the complete spectrum from technical proficiency to professional character.

(1) Knowledge

Researchers have extensively investigated the knowledge required by quantity surveyors, identifying numerous elements critical to their professional performance. Yang (2013) [26] emphasized on core understanding of civil engineering, engineering cost management, economic theories, and legal frameworks. Yogeshwaran et al., Chamikara, and Roztocki et al. all highlighted the necessity of construction information technology (IT) and business administration capabilities [7,27,28]. Chamikara et al. stressed the importance of knowledge in building technology, environmental services, computer literacy, and measuring and costing [27]. According to Arun et al., critical competencies include contract documentation, construction technology, project management, and cost estimation, as well as skills in dispute resolution, life cycle cost analysis, budgetary processes, value management, and computer and information technology [10]. Agnieszka et al. stated that quantity surveyors must be proficient in computer tools, science, mathematics, engineering concepts, and information technology applications [24].

Consequently, through integration, the essential QS knowledge in this study is represented by eight competency indicators: construction economics, construction management, construction technology, construction law, construction information technology, business administration, measurement and costing, and contract administration.

(2) Skills

Several scholars have investigated the critical skills required for quantity surveyors. Yang emphasized organizational flexibility, collaborative skills, planning, organizing, coordinating, regulating, and decision-making abilities as critical for quantity surveyors [26]. Yogeshwaran et al.and Chamikara et al. highlighted the importance of teamwork, leadership, communication, presenting skills, client care, and leadership and management abilities, respectively [7,27]. According to Arun et al., key skills for quantity surveyors include analytical skills, professional practice, quantification/measurement, personal and interpersonal skills, leadership, business and management skills, communication skills, and manual skills [10]. Agnieszka et al. identified communication, written expression, working effectively with people, addressing real-world challenges, and cooperation as crucial for quantity surveyors [24]. Furthermore, John and Clinton underscored the importance of communication, personal skills, time management, managerial abilities, and analytical skills for quantity surveyors [29].

Consequently, the key QS skills identified through integration are teamwork skills, communication skills, judgement and decision making skills, manual skills, and reporting skills.

(3) Ethics

Several scholars have investigated significant ethical considerations for quantity surveyors. Linda et al. identified duty, fairness, categorical imperative, competence, responsibility, and a commitment to serve the public as critical ethical concepts for quantity surveyors [30]. Yang emphasized honesty, integrity, adherence to rules and regulations, commercial secrecy, and concern for the public good and social responsibility [26]. Yogeshwaran et al. and Chamikara et al. highlighted the importance of ethics and professional behaviour, value management, and fairness in quality assurance practice [7,27]. Lian and Ling stressed fairness in business, while Akure underscored trustworthiness, responsibility, and loyalty as ethical imperatives [17,31]. Agnieszka et al. broadened the scope to include engineering ethics, personal ethics and value, environmental and global awareness, and contributing to society and the community as important ethical issues for quantity surveyors [24].

Consequently, the core ethical principles for QS, derived through integration, are primarily social responsibility, justice, and professional morals.

(4) Personality

Consequently, personality significantly influences work performance in the construction industry. Individual differences among quantity surveyors affect their job satisfaction [17]. The literature review indicates that personality encompasses multiple facets. Lian and Ling emphasized factors such as job enthusiasm, workload management, task diversity, promotion opportunities, and workplace fairness in the QS context [17]. Elvy et al. expanded on this, identifying attributes like adaptability, initiative, self-confidence, self-control, creativity, innovation, the capacity and desire to learn, and analytical thinking as critical for success in the field [32]. Manikandan highlighted personal appearance and attributes that shape professional image [22]. According to Agnieszka et al. and John & Clinton, quantity surveyors must be motivated, creative, enthusiastic, and possess specific personal qualities [24,29].

Consequently, the fundamental personality attributes for QS, derived through this integration, are primarily resilience, professional initiative, innovative insight, and personal image.

According to prior research and analysis, a comprehensive review of extant literature on quantity surveying competencies, graduate attributes, and industry expectations was conducted, generating a preliminary list of potential competency items. To condense this list into a manageable and non-redundant set of indicators, the strategies of conceptual clustering and thematic synthesis were employed. The final selection of the 20 indicators was guided by the primary criteria: The indicator must be explicitly or implicitly cited in literature as a key expectation for entry-level quantity surveyors. A detailed mapping between the original literature constructs and the final 20 QS competency indicators is provided in Table 2.

**Table 2:** Mapping of original literature constructs to final 20 QS competency indicators. Source: own research.

Competency domains	Final competency indicators	Original competency items	Author
Knowledge	Construction economics	economic theories, value management	Yang; Arun
	Construction management	project management	Arun
	Construction technology	building technology,construction technology,	Chamikara et al.; Arun
	Construction law	skills in dispute resolution	Arun
	Construction information technology	construction information technology (IT),computer literacy,computer and information technology,computer tools,information technology applications	Yogeshwaran et al.; Chamikara et al.; Roztocki et al.; Arun; Agnieszka, et al.
	Business administration	business administration capabilities	Yogeshwaran et al.; Chamikara et al.; Roztocki et al.
	Measurement and costing	engineering cost management,measuring and costing,cost estimation,life cycle cost analysis, budgetary processes,	Yang; Chamikara et al.; Arun
	Contract administration	contract documentation,	Arun
Skills	Teamwork skills	organisational flexibility,collaborative skills, planning, organising,coordinating, regulating,teamwork,working effectively with people,cooperation,	Yang; Yogeshwaran et al.; Chamikara et al.; Agnieszka et al.
	Communication skills	communication,personal and interpersonal skills,communication skills,	Yogeshwaran et al.; Chamikara et al.; Arun et al.; Agnieszka et al.; John & Clinton)
	Judgment and decision making skills	decision-making abilities,leadership and management abilities, leadership,analytical skills,	Yang; Yogeshwaran et al.; Chamikara et al.; Arun et al.; Agnieszka et al.
	Manual skills	professional practice, quantification/measurement, manual skills	Arun et al.
	Reporting skills	presenting skills, written expression	Yogeshwaran et al.; Chamikara et al.; Agnieszka et al.
Personality	Resilience	workload, task variety, flexibility	Lian & Ling; Elvy et al.
	Professional initiative	passion for the job, advancement opportunity, initiative, self-confidence, self-control, willingness to learn, motivation, enthusiasm	Lian & Ling; Elvy et al.; John & Clinton
	Innovative insight	creativity, innovation and change, analytical thinking	Elvy et al.; John & Clinton
	Personal image	characteristics, personal image	Manikandan
Ethics	Social responsibility	duty, responsibility, willingness to serve the public, concern for public interests and social responsibility, responsibility, understanding of environmental concerns and understanding of global concerns, contributing to society and the community, global and social content	Linda et al.; Yang; Akure; Agnieszka et al.
	Justice	justice, honesty, fairness, personal ethics and valued	Linda et al.; Yang; Yogeshwaran et al.; Lian & Ling; Agnieszka et al.
	Professional moral	integrity, compliance with law and regulations, preservation of commercial confidentiality, ethics and professional conduct, ethics and professional practice, trustworthiness, loyalty, engineering ethics	Yang; Yogeshwaran et al.; Chamikara et al.; Akure; Agnieszka et al.

### Conceptual framework and hypothesis

The integration of the Onion Model and the MULFR approach is theoretically justified by the P-E Fit framework. The Onion Model provides the essential conceptual structure by decomposing the holistic concepts of “graduate” and “industry” into a layered, multidimensional set of specific, measurable competency indicators (e.g., from inner-layer “Personal Image” to outer-layer “Technical Skills”). This decomposition is a prerequisite for any meaningful measurement of fit. Subsequently, the MULFR model provides the statistical engine to rigorously quantify the P-E fit. Traditional statistical methods often struggle with measurement error and the high-dimensional nature of competency data. The MULFR model, however, is specifically designed to model the functional relationship between two sets of multidimensional variables (Graduate Competencies and Industry Expectations), each measured with inherent error via questionnaires. Therefore, while the Onion Model defines what is being compared, the MULFR model defines how the comparison is executed with statistical rigor, together offering a comprehensive solution to the problem of measuring competency gaps within the P-E Fit paradigm.

**Fig 2:**
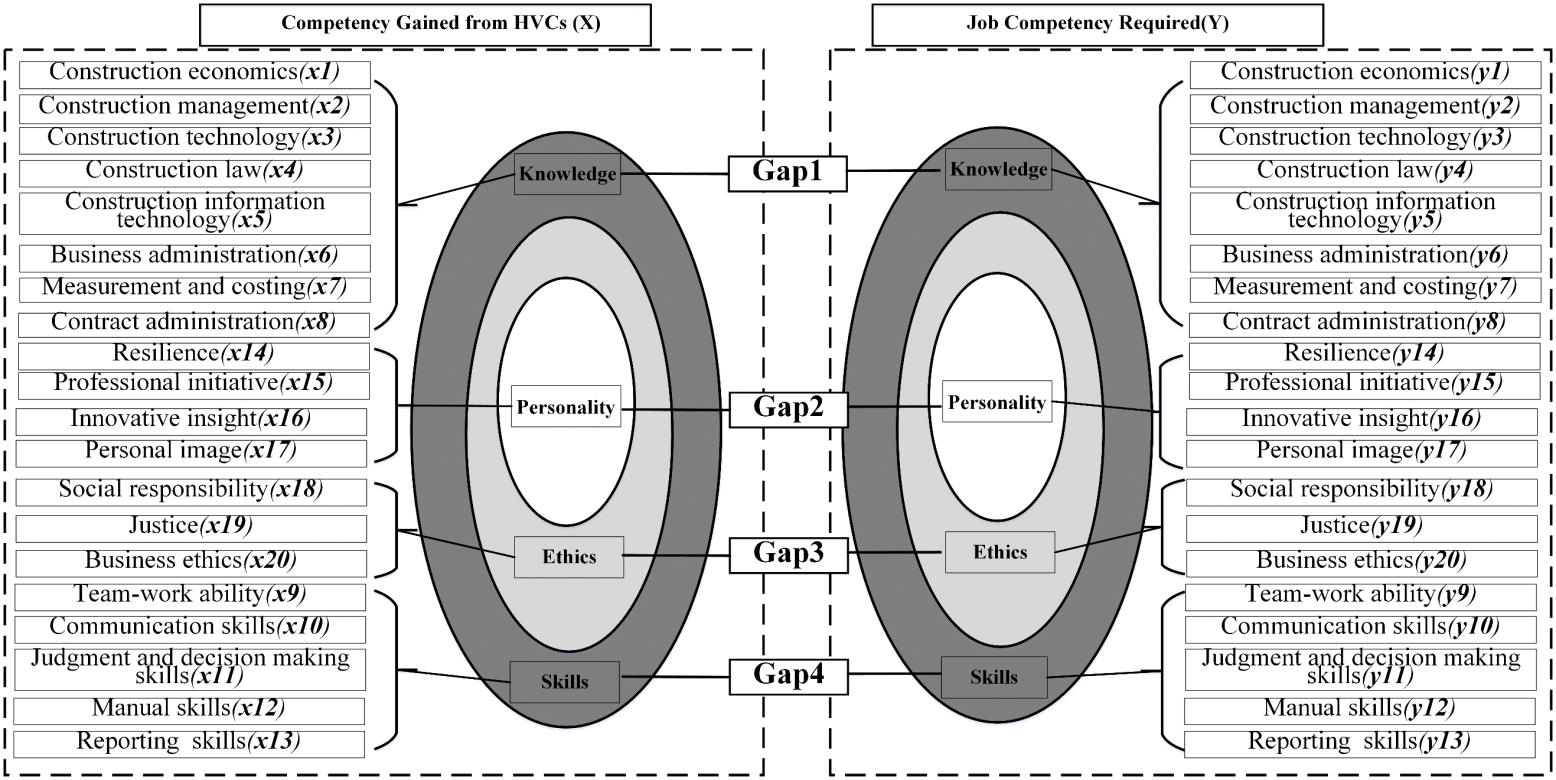
Conceptual framework.

This comprehensive analysis considered the issues associated with the QS competency gap, as well as the relevant theories identified in the literature review. The study integrated these elements, establishing a solid foundation and providing a basis for hypothesis development. Consequently, the following conceptual framework was developed based on the theoretical foundation and key findings. Fig 2 presents this conceptual framework.

Grounded in the Person-Environment Fit Theory [22] and prior evidence of industry-education misalignment in China [1], this study proposes the following hypotheses. Rather than merely testing for the existence of gaps, these hypotheses predict the specific nature of the discrepancy based on the documented over-reliance on theoretical knowledge and under-emphasis on managerial and soft skills in HVCs.

H1: A gap exists in the knowledge domain, wherein the industry’s expectation for technical and regulatory knowledge surpasses the perceived competency of HVC graduates.

H2: A gap exists in the skills domain, specifically in applied technical skills, where industry requirements are predicted to exceed the proficiency level of recent graduates.

H3: There will be no gap in the ethics domain, as ethical principles are a foundational and consistently emphasized component of both HVC curricula and professional practice.

H4: A gap exists in the personality domain, reflecting a systemic underdevelopment of these attributes within the current practice-oriented HVC training model.

## Materials and methods

### Sample size

This study employed a quantitative questionnaire survey with a purposive sampling technique to analyze and identify competency gaps among recent higher vocational diploma graduates in quantity surveying. The target population consisted of recent QS graduates who completed their studies at HVCs in China and had worked in the QS industry for no more than three years. The data were accessed for research purposes on 10th October, 2023. And the study adhered to ethical standards and ensured participant anonymity and confidentiality. All experimental protocols were approved by Dianchi College Scientific Research Office. All participants were provided with a detailed digital information sheet before the survey, which outlined the study’s purpose, data usage, and their rights. Participation was entirely voluntary, and respondents indicated their electronic consent by proceeding to the questionnaire. No personally identifiable information was collected at any stage. The survey data were anonymized upon submission, and all analyses were performed on aggregated, non-identifiable data.The collected anonymized data were stored on a secure, password-protected server accessible only to the research team.

As of 2022, China had approximately 578 HVCs offering QS majors, producing around 80,342 QS graduates. A portion of these graduates, who had been employed in QS-related roles for less than three years, constituted the sampling frame for our study.

Several factors are considered when determining the sample size. Random sampling often follows established statistical formulas, such as those proposed by Krejcie and Morgan, who recommend a minimum sample size of 385 for a 95% confidence level [33]. Previous research has utilized various sample sizes, including studies by Chamikara et al.with 79 participants, and Agnieszka et al. and Arun et al., each with approximately 250 participants [10,24,27]. To minimize sampling error and ensure data robustness, this study recruited 350 recent QS graduates for the questionnaire survey, from which 324 complete responses were obtained.

### Measurement tool and data collection

The data collection instrument was a questionnaire designed to assess the gap between industry expectations and graduates’ current competencies. These respondents answered to questions on the competencies they learnt in school and the skill sets necessary for their careers. The survey employed a purposive sampling method. According to Johnson & Smith, purposive sampling can be advantageous in various research scenarios, including exploratory studies or investigations involving specific subgroups within a larger population [9].The following describes the procedure for purposive sampling: (a) HVCs in China which offer QS programmes are identified, and their permission is obtained to help send the questionnaire to their QS alumni who graduated. (b) All QS graduates (respondents) receiving the questionnaire are required to answer two qualifying questions: (i) whether they graduated from the QS programme, and (ii) they have no more than three years of QS related working experience. Respondents who failed to fulfil these two conditions will be excluded from this study.

Data were collected via an online questionnaire distributed through professional networks, and alumni associations of several HVCs. A total of 324 valid responses were obtained. While this sample size is adequate for the proposed MULFR model analysis, it is crucial to acknowledge that the purposive sampling method means the sample is not statistically representative of the entire national population of QS HVC graduates. The findings, therefore, are primarily analytically generalizable; they provide a validated conceptual framework and identify critical areas of concern that are likely relevant in similar contexts, rather than yielding precise population parameter estimates. The potential for self-selection bias was mitigated by ensuring anonymity and emphasizing the study’s academic purpose in the recruitment materials. However, it is important to note that while this method enriches the variety within the sample, it does not aim to achieve statistical representativeness of the entire population of Chinese quantity surveying graduates.

The questionnaire was divided into two sections and utilized a five-point Likert scale with responses ranging from 1 (strongly disagree) to 5 (strongly agree). Section A collected demographic information, including gender, educational background, and current employment position, as detailed in Table 3. Section B assessed core competency components—knowledge, skills, ethics, and personality traits—which were adapted from established literature.

**Table 3:** Research on QS competency standards. Source: own research.

Question	Answer	N	Valid percent (%)	Cumulative percent (%)
Gender	Male	150	46.3	46.3
	Female	174	53.7	100.0
Education level	Higher vocational diploma	324	100	100.0
Current job position type	QS consulting	74	22.8	22.8
	Business and technology	51	15.7	38.5
	Bidding and procurement	44	13.6	52.1
	Financial auditing	24	7.4	59.5
	Clerical and administrative	12	3.7	63.2
	Investment management	24	7.4	70.6
	Project management	43	13.3	83.9
	Education/training	30	9.3	93.2
	Legal consulting	21	6.5	99.7
	Other related	1	0.3	100

Of the 324 respondents, 53.7% were female (n = 174) and 46.3% were male (n = 150). In terms of educational background, all 324 respondents had obtained a higher vocational diploma from an HVC. Regarding current employment, QS consulting had the highest representation (22.8%), followed by business and technology (15.7%), bidding and procurement (13.6%), and project management (13.3%). These roles were significantly more prevalent than other career paths.

### Competency gap measure using $R^{2}$ from MULFR model

This work evaluates the competency gap using a novel statistical technique, the MULFR model [4]. Although the MULFR model has been developed since 2010, this study is the first attempt to apply it to competency gap analysis, as previous measures do not account for multidimensional domains and indicators in competency. (a) The other methods, such as Person correlations, simple linear regressions, the mean rank method [10], hierarchical analysis, Bloom’s Taxonomy [7], the Mann-Whitney U test [18], and structural equation modelling (SEM) [14]. there is only one variable ***Y*** with multiple variables ***X***. In my case, we need to compare multiple variables ***Y*** and multiple variables ***X*** as a single model. (b) In linear regression model, the response (dependent) variable ***Y*** is subject to error, while the independent variable ***X*** is fixed, means that there is no error. However, MULFR model is an extension of the classical linear regression model. In the MULFR model, with expanded assumptions, both the response (dependent) variable ***Y*** and independent variable ***X*** are subject to error. (c) With the other methods, some studies may want to understand the relationship or similarity between a set of multidimensional variables and another set of multidimensional variables. The utilization of bivariate correlation measures by averaging the correlation coefficients between all possible pairs tends to underestimate the relationship between multidimensional ***X*** and ***Y***. In the MULFR model, we can put all the variables into the same model, and we can get one competency gap, the $R^{\text{2}}$ value.

In this case, we need to compare multiple variables Y and multiple variables Xas a single model. The MULFR model is a suitable and reliable measure for competency gap analysis. For the primary purpose of this study is to compare the post competency gaps between industry expectations and the current competencies of graduated QSs from HVCs in China. MULFR model is an extension of the conventional multiple linear regression model by comparing two sets of p-dimensional variables subject to measurement errors. And this model can solve the problem properly.

In this context, $Y_{\text{1}},Y_{\text{2}},\ldots ,Y_{p}$ correspond to separate competency domains or dimensions required by industry, whereas $X_{\text{1}},X_{\text{2}},\ldots ,X_{p}$ reflects the same set of competency domains or dimensions obtained through HVCs. Assume $Y_{i}={{(Y}_{\text{1}i},Y_{\text{2}i},{\ldots ,Y}_{pi})}^{\prime }$ and $X_{i}={{(X}_{\text{1}i},X_{\text{2}i},{\ldots ,X}_{pi})}^{\prime }$ are two linearly linked unobservable true values of two variables with *p*-dimensions such that


$$Y_{i}=\;\alpha +\beta X_{i},\;i=\text{1},\ldots ,n$$ (1)

where $\alpha ={{(\alpha }_{\text{1}},\alpha_{\text{2}},{\ldots ,\alpha }_{p})}^{\prime }$ represents the linear function’s intercepts, whereas β represents its slope. The random vectors $y_{i}={{(y}_{\text{1}i},y_{\text{2}i},{\ldots ,y}_{pi})}^{\prime }$ and $x_{i}={{(x}_{\text{1}i},x_{\text{2}i},{\ldots ,x}_{pi})}^{\prime }$ are observed with errors $\;\delta_{i}={{(\delta }_{\text{1}i},\delta_{\text{2}i},{\ldots ,\delta }_{pi})}^{\prime }$ and $\varepsilon_{i}={{(\varepsilon }_{\text{1}i},\varepsilon_{\text{2}i},{\ldots ,\varepsilon }_{pi})}^{\prime }$ such that,


$$\;\begin{array}{c} x_{i}=X_{i}+\delta_{i}\\ y_{i}=Y_{i}+\varepsilon_{i} \end{array}\}i=\text{1},\text{2},\ldots ,n$$ (2)

Variables ***X*** and ***Y*** are scores provided by QS graduates, especially in competency gap analysis, on various competency characteristics obtained via HVCs and necessary on the job. Given the mutual and independent normal distributions of both error vectors,

(i) $\text{E}\left(\delta_{\text{i}}\right)=\text{0}=\text{E}\left(\epsilon_{\text{i}}\right)$
(ii) $\text{var}\left(\delta_{\text{ki}}\right)=\sigma^{\text{2}\;}$ and $\;\;\;\text{var}\left(\epsilon_{\text{ki}}\right)=\tau^{\text{2}\;}$ for k=1,…,p. i=1,…,n
(iii) $\text{cov}\left(\delta_{\text{ki}},\delta_{\text{kj}}\right)=\text{0}=$ $\text{var}\left(\epsilon_{\text{ki}},\epsilon_{\text{kj}}\right)$, for all i≠j.i,j=1,…,n
$\text{cov}\left(\delta_{\text{ki}},\delta_{\text{hi}}\right)=\text{0}=$ $\text{var}\left(\epsilon_{\text{ki}},\epsilon_{\text{hi}}\right)$, for all h≠k.h,k=1,…,p,i=1,…,n
and $\text{cov}\left(\delta_{\text{ki}},\epsilon_{\text{hj}}\right)=\text{0}$, for all i,j=1,…,n  and h,k=1,…,p

that is $\delta_{i}\sim IND(0,\Omega_{\text{2}\text{2}}$) and $\varepsilon_{i}\sim IND(0,\Omega_{\text{1}\text{1}}$) where $\Omega_{\text{1}\text{1}}=[\begin{array}{ccc} \tau^{\text{2}} & \cdots & \text{0}\\ \vdots & \ddots & \vdots \\ \text{0} & \cdots & \tau^{\text{2}} \end{array}]=\tau^{\text{2}}I,$ $\Omega_{\text{2}\text{2}}=[\begin{array}{ccc} \sigma^{\text{2}} & \cdots & \text{0}\\ \vdots & \ddots & \vdots \\ \text{0} & \cdots & \sigma^{\text{2}} \end{array}]=\sigma^{\text{2}}I$ and let $v_{i}=(\begin{array}{c} \varepsilon_{i}\\ \delta_{i} \end{array})$, then $cov\left(v_{i},v_{i}\right)=\Omega =[\begin{array}{cc} \Omega_{\text{1}\text{1}} & \Omega_{\text{1}\text{2}}\\ \Omega_{\text{2}\text{1}} & \Omega_{\text{2}\text{2}} \end{array}]$ are diagonal variance-covariance matrices, $\Omega_{\text{1}\text{2}}=\Omega_{\text{2}\text{1}}=0,$ $\Omega_{\text{1}\text{1}}$ and $\Omega_{\text{2}\text{2}}$ are positive definite.

The estimated slope coefficient is


$$\hat{\beta }=\frac{-(\lambda S_{xx}-S_{yy})\pm \sqrt{({\lambda S_{xx}-S_{yy})}^{\text{2}}+\text{4}\lambda S_{xy}^{\text{2}}}}{\text{2}S_{xy}}$$ (3)

$$\alpha =\;\overset{―}{y}-\beta \overset{―}{x}$$ (4)

where λ = 1 is the ratio of error variances, and $S_{xx}=\sum_{i=\text{1}}^{n}x_{i}^{{}^{\prime }}x_{i}-n\overset{―}{x^{{}^{\prime }}}\overset{―}{x}$, $S_{yy}=\sum_{i=\text{1}}^{n}y_{i}^{{}^{\prime }}y_{i}-n\overset{―}{y^{{}^{\prime }}}\overset{―}{y}$, and $S_{xy}=\sum_{i=\text{1}}^{n}x_{i}^{{}^{\prime }}y_{i}-n\overset{―}{x^{{}^{\prime }}}\overset{―}{y}$.

The coefficient of determination of the MULFR model is


$$R_{p}^{\text{2}}=\frac{{SS}_{R}}{S_{yy}}=\frac{{\hat{\beta }S}_{xy}}{S_{yy}}\;\;$$ (5)

The quantity $R_{p}^{\text{2}}$ in Equation (5) measures the similarity between the QS competency expected by industry and competency acquired from HVCs. Hence, $\text{1}-R_{p}^{\text{2}}$ measure the competency gap reflected on the fresh graduated quantity surveyors.

In this study, a higher $R_{p}^{\text{2}}$ value suggests better alignment between school-based capabilities and industrial requirements, and vice versa. Table 4 summarizes the criteria for determining the skill gap, which have been adapted from Cohen [34]. Additionally, two values of $R_{p}^{\text{2}}$ with at least 5% difference indicates a substantial difference, whilst values below this level are regarded inconsequential.

**Table 4:** Criteria for competency gap using $R_{p}^{2}$.

$R_{p}^{2}$	$1-R_{p}^{2}$	Interpretation
[0,0.65)	(0.35,1.0]	large gap
[0.65,0.8)	(0.2,0.35]	medium gap
[0.8,0.9)	(0.1,0.2]	small gap
[0.9,1.0]	[0,0.1]	no gap

Value differences between $R_{p}^{\text{2}}$≥ 5%, significant, or else is not significant.

### Reliability and validity analysis of questionnaire data

The reliability and validity of the questionnaire data were assessed using SPSS 26.0 prior to the main analysis. Reliability was evaluated using Cronbach’s alpha to measure internal consistency. The results demonstrated excellent reliability, with an overall Cronbach’s alpha of 0.965 (95% CI [0.959, 0.970]). The reliability for each competency dimension was also high, with all alpha coefficients exceeding 0.85 (see Table 5 for full details). Furthermore, item-total correlations for all indicators were above the recommended threshold of 0.5, and no deletion of any item would have substantially improved the alpha value for its respective dimension, confirming the robustness of the scale.

**Table 5:** Reliability and validity statistics on the competency indicator groups of each dimension.

Domains	Indicators	Cronbach’s alpha	KMO	Sig.
	**Total**	**0.965**	**0.876**	**.000**
Knowledge	Construction economics	0.904	0.953	.000
	Construction management	0.902	0.960	.000
	Construction technology	0.881	0.962	.000
	Construction law	0.885	0.962	.000
	Construction information technology	0.901	0.956	.000
	Business administration	0.861	0.963	.000
	Measurement and costing	0.919	0.959	.000
	Contract administration	0.911	0.956	.000
Skills	Teamwork skills	0.922	0.921	.000
	Communication skills	0.931	0.917	.000
	Judgment and decision making skills	0.929	0.815	.000
	Manual skills	0.897	0.890	.000
	Reporting skills	0.896	0.887	.000
Personality	Resilience	0.885	0.891	.000
	Professional initiative	0.899	0.917	.000
	Innovative insight	0.882	0.839	.000
	Personal image	0.867	0.830	.000
Ethics	Social responsibility	0.924	0.911	.000
	Justice	0.885	0.888	.000
	Professional moral	0.903	0.893	.000

Validity, specifically structural validity, was examined through Exploratory Factor Analysis (EFA). The sampling adequacy was verified by a Kaiser-Meyer-Olkin (KMO) measure of 0.876 for the entire set of twenty competency indicators, and Bartlett’s test of sphericity was significant, p < 0.001, supporting the factorability of the data. A principal component analysis with Varimax rotation was conducted. The first factor’s eigenvalue is about 31.215, representing a variance contribution of 23.295%, explaining a significant portion of the 134 variables. This factor is the principal component with the highest variance contribution. The top 20 factors collectively explain 85.325% of all variables, with eigenvalues exceeding 1 and 20 common factors can be identified in this scenario. All factor loadings for the retained items were greater than 0.60, with the majority exceeding 0.70, confirming that each item adequately loaded onto its intended theoretical construct.

## Results

The MULFR model was employed in this study to calculate gap values for 20 competency indicators across four domains: knowledge, skills, personality, and ethics. This method enables a comprehensive assessment of competency gaps, revealing the discrepancies between the competency levels attained through academic education and those demanded by employers. By applying the MULFR model, the study quantifies competency gap values through a comparison of the competencies possessed by recent QS graduates and those required by the construction industry. The analysis is based on survey data and addresses the following competency domains: knowledge, skills, personality, and ethics.

Table 6 presents the $R_{p}^{\text{2}}$ and ${(\text{1}-R}_{p}^{\text{2}\;})$ results, including the competency gap values across the four domains and 20 indicators. These findings highlight the disparities between the current skill levels of higher vocational diploma QS graduates and the expectations of the construction industry, offering insights for targeted development to effectively bridge these gaps.

**Table 6:** Gap values of the 4 domains and 20 competency indicators.

Competency domains	$R_{p}^{2}$ $(1-R_{p}^{2}$)	Competency indicators	Vector	$R_{p}^{2}$ $(1-R_{p}^{2}$)
Knowledge	0.81 (0.19)	Construction economics	$x_{\text{1}}$	0.82
			$y_{\text{1}}$	(0.18)
		Construction management	$x_{\text{2}}$	0.81
			$y_{\text{2}}$	(0.19)
		Construction technology	$x_{\text{3}}$	0.78
			$y_{\text{3}}$	(0.22)
		Construction law	$x_{\text{4}}$	0.80
			$y_{\text{4}}$	(0.20)
		Construction information technology	$x_{\text{5}}$	0.82
			$y_{\text{5}}$	(0.18)
		Business administration	$x_{\text{6}}$	0.74
			$y_{\text{6}}$	(0.26)
		Measurement and costing	$x_{\text{7}}$	0.85
			$y_{\text{7}}$	(0.15)
		Contract administration	$x_{\text{8}}$	0.83
			$y_{\text{8}}$	(0.17)
Skills	0.84 (0.16)	Teamwork skills	$x_{\text{9}}$	0.85
			$y_{\text{9}}$	(0.15)
		Communication skills	$x_{\text{1}\text{0}}$	0.87 (0.13)
			$y_{\text{1}\text{0}}$	
		Judgment and decision making skills	$x_{\text{1}\text{1}}$	0.86
			$y_{\text{1}\text{1}}$	(0.14)
		Manual skills	$x_{\text{1}\text{2}}$	0.82
			$y_{\text{1}\text{2}}$	(0.18)
		Reporting skills	$x_{\text{1}\text{3}}$	0.82
			$y_{\text{1}\text{3}}$	(0.18)
Personality	0.78 (0.22)	Resilience	$x_{\text{1}\text{4}}$	0.81
			$y_{\text{1}\text{4}}$	(0.19)
		Professional initiative	$x_{\text{1}\text{5}}$	0.81
			$y_{\text{1}\text{5}}$	(0.19)
		Innovative insight	$x_{\text{1}\text{6}}$	0.78
			$y_{\text{1}\text{6}}$	(0.22)
		Personal image	$x_{\text{1}\text{7}}$	0.75
			$y_{\text{1}\text{7}}$	(0.25)
Ethics	0.82 (0.18)	Social responsibility	$x_{\text{1}\text{8}}$	0.86
			$y_{\text{1}\text{8}}$	(0.14)
		Justice	$x_{\text{1}\text{9}}$	0.79
			$y_{\text{1}\text{9}}$	(0.21)
		Professional moral	$x_{\text{2}\text{0}}$	0.81
			$y_{\text{2}\text{0}}$	(0.19)

### Competency gap analysis based on the four domains

Across the HVCs, the $R_{p}^{\text{2}}$ value for “Personality” is 0.78, the lowest among the four competency domains. It suggests that the competency level developed in “Personality” within HVC is the least aligned with enterprise competency requirements. And $\left(\text{1}-R_{p}^{\text{2}}\right)$=0.22, indicating a medium competency gap. The $R_{p}^{\text{2}}$ value for “Skills” is 0.84, the highest among the four competency domains. It implies that the “Skills” competency developed in HVCs is the most aligned with enterprise competency requirements. And $\left(\text{1}-R_{p}^{\text{2}}\right)$=0.16, indicating a small competency gap. Additionally, the discrepancy in $R_{p}^{\text{2}}$ values between “Personality” ($R_{p}^{\text{2}}$ = 0.78) and “Skills” ($R_{p}^{\text{2}}$ = 0.84) is 6%.

Therefore, for all the HVCs, a medium competency gap exists in the “Personality” domain between the competency level developed through academic education and enterprise requirements, while small competency gaps are observed in “Skills”, “Knowledge”, and “Ethics”.

In summary, these findings are consistent with prior studies by Liu et al. and Smith & Johnson, which also identified a misalignment between the personality traits cultivated during HVC education and those required by the industry for new quantity surveyors [1,9]. Furthermore, as indicated in Brown & Lee’s study, this gap suggests that the personality traits emphasized in the school curriculum may not adequately address the practical demands and expectations of the quantity surveying industry [35].

### Competency gap analysis for knowledge

The $R_{p}^{\text{2}}$ value for “Business administration” is 0.74, the lowest among all indicators. This suggests that the competency level in “Business administration” knowledge developed in HVCs is the least able to meet enterprise requirements. And $\left(\text{1}-R_{p}^{\text{2}}\right)$=0.26, indicating a medium competency gap. The $R_{p}^{\text{2}}$ value for “Measurement and costing” is 0.85, the highest among all indicators. It implies that the competency level in “Measurement and costing” knowledge aligns most closely with enterprise requirements. And $\left(\text{1}-R_{p}^{\text{2}}\right)$=0.15, signaling a small competency gap. Additionally, the discrepancy in $R_{p}^{\text{2}}$values between “Business administration” ($R_{p}^{\text{2}}$ = 0.74) and “Measurement and costing” ($R_{p}^{\text{2}}$ = 0.85) is 11%.

Therefore, for all the HVCs, medium competency gaps exist between the knowledge developed through academic education and enterprise requirements in “Business administration”, and “Construction technology”, while small competency gaps are observed in “Construction economics”, “Construction management”, “Construction law”, “Construction information technology”, “Measurement and costing”, and “Contract administration”.

In conclusion, these findings corroborate those of Yogeshwaran et al., suggesting that while business administration is not a core technical competency for QS, it is highly valued and expected by the industry [7]. According to the survey results, 67.9% of QS graduates reported a lack of exposure to this course or related knowledge, indicating a significant gap in their training. Consequently, while organizations have a substantial demand for business administration knowledge, a notable gap exists in its supply among graduates. Overall, these findings are consistent with Agnieszka et al., who also reported high alignment between the knowledge areas learned in school and those required by employers [24].

### Competency gap analysis for skills

The $R_{p}^{\text{2}}$ value for both “Manual skills” and “Reporting skills” is 0.82, the lowest among the indicators. It suggests that competency levels in these two areas are the least aligned with enterprise requirements. And $\left(\text{1}-R_{p}^{\text{2}}\right)$=0.18, indicating a small competency gap. Conversely, the $R_{p}^{\text{2}}$ value for “Communication skills” is 0.87, the highest among the indicators. It implies that competency levels in communication skills align most closely with enterprise requirements. And $\left(\text{1}-R_{p}^{\text{2}}\right)$=0.13, also indicating a small competency gap. Additionally, the discrepancy in $R_{p}^{\text{2}}$ values between “Manual skills” & “Reporting skills” ($R_{p}^{\text{2}}$ = 0.82) and “Communication skills” ($R_{p}^{\text{2}}$ = 0.87) is 5%.

Consequently, all five skills – “Teamwork skills,” “Communication skills,” “Judgment and decision-making skills,” “Manual skills,” and “Reporting skills” – exhibit small competency gaps due to disparities between HVC education and industry demands.

In conclusion, these findings align with those of Yogeshwaran et al., who emphasized the importance of skills in resource analysis and the budgeting process for QS graduates and identified corresponding educational gaps [7]. This study’s results, showing small but consistent gaps across all skill domains, partially reflect the sentiment reported by Agnieszka et al., wherein respondents rated various skill categories as highly important to enterprises [24]. Furthermore, the specific skill gaps observed resonate with Yogeshwaran et al.’s emphasis on the critical role of key support abilities such as teamwork, leadership, communication, presentation, and client service in the QS profession [7].

### Competency gap analysis for personality

The $R_{p}^{\text{2}}\;$ value for “Personal image” is 0.75, the lowest among the indicators. It suggests that the competency level for “Personal image” developed in HVCs is the least aligned with enterprise requirements. And $\left(\text{1}-R_{p}^{\text{2}}\right)$=0.25, indicating a medium competency gap. Conversely, the $R_{p}^{\text{2}}\;$ value for both “Resilience” and “Professional initiative” is 0.81, the highest among the indicators. It implies that the competency levels for “Resilience” and “Professional initiative” align most closely with enterprise requirements. And $\left(\text{1}-R_{p}^{\text{2}}\right)$=0.19, indicating a small competency gap. Additionally, the discrepancy in $R_{p}^{\text{2}}\;$ values between “Personal image” ($R_{p}^{\text{2}}\;$ = 0.75) and “Resilience” & “Professional initiative” ($R_{p}^{\text{2}}$ = 0.81) is 6%.

Consequently, “Innovative insight” and “Personal image” exhibit medium competency gaps, whereas “Resilience” and “Professional initiative” show small competency gaps.

In conclusion, the findings regarding the small gaps in “Resilience” and “Professional initiative” are consistent with John and Clinton’s report that QS professionals generally experience good job satisfaction and appreciate diverse responsibilities [29]. However, the observed medium gaps in “Innovative insight” and “Personal image” align with the findings of Liu et al. and Smith & Johnson, who identified a misalignment between the personality traits cultivated during HVC education and those demanded by the industry [1,9]. Furthermore, as suggested by Brown and Lee, this specific misalignment indicates that the personality traits emphasized in the school curriculum may not fully address the practical demands and requirements of the quantity surveying profession [35].

### Competency gap analysis for ethics

The $R_{p}^{\text{2}}$ values for “Justice” is 0.79, the lowest among the ethics indicators. It suggests that the competency level for “Justice” developed in HVCs is less aligned with enterprise requirements. And $\left(\text{1}-R_{p}^{\text{2}}\right)$=0.21, indicating a medium competency gap. Conversely, the $R_{p}^{\text{2}}$ value for “Social responsibility” is 0.86, the highest among the indicators. It implies that the competency level for “Social responsibility” aligns most closely with enterprise requirements. And $\left(\text{1}-R_{p}^{\text{2}}\right)$=0.14, indicating a small competency gap. Additionally, the discrepancy in $R_{p}^{\text{2}}$ values between “Justice” ($R_{p}^{\text{2}}$ = 0.79) and “Social responsibility” ($R_{p}^{\text{2}}$ = 0.86) is 7%.

Therefore, for HVCs, a medium competency gap exists in “Justice”, while small competency gaps are observed in the other two indicators, “Social responsibility” and “Professional moral”.

In conclusion, these findings align with Yogeshwaran et al., who identified ethics, professional conduct, and value management as essential traits for QS graduates, noting a significant gap between educational preparation and industry requirements [7]. The medium gap in “Justice” observed in this study resonates with Linda et al., who discovered variations in the understanding of ethical norms among professional QSs[30]. This study’s data further indicate that respondents demonstrated a strong inclination toward the ethical principle of “Justice”. Collectively, these findings corroborate and refine prior research outcomes, confirming a consistent pattern while providing more granular insight into specific ethical competencies.

## Discussion

Using the MULFR model, the study identified competency gaps between the performance of QS graduates from HVCs and industry standards. A summary of the key competency gap findings across domains and for the indicators with the largest gaps is provided in Table 7. The “Personality” domain exhibited the largest competency gap $\left({\text{1}-R}_{p}^{\text{2}}=\text{0}.\text{2}\text{2}\right)$, whereas the “Skills” domain showed the smallest gap $\;\left({\text{1}-R}_{p}^{\text{2}}=\text{0}.\text{1}\text{6}\right)$. Table 7 displays the top three competency gap indicators: “Business administration” $\;\left({\text{1}-R}_{p}^{\text{2}}=\text{0}.\text{2}\text{6}\right)$, “Personal image” $\;\left({\text{1}-R}_{p}^{\text{2}}=\text{0}.\text{2}\text{5}\right)$, and “Innovative insight” $\;\left({\text{1}-R}_{p}^{\text{2}}=\text{0}.\text{2}\text{2}\right)$.

**Table 7:** Summary of competency gap findings across domains and key indicators.

Rank	Competency Domain/ Indicator	$R_{p}^{2}$	${1-R}_{p}^{2}$	Gap Magnitude
**By Domain**				
1(Largest)	**Personality**	**0.78**	**0.22**	Medium
2	Knowledge	0.81	0.19	Small
3	Ethics	0.82	0.18	Small
4(Smallest)	**Skills**	**0.84**	**0.16**	Small
**By Indicator(Top 3)**				
1	Business administration	0.74	0.26	Medium
2	Personal image	0.75	0.25	Medium
3	Innovative insight	0.78	0.22	Medium

The gap in business administration competencies likely stems from a curriculum that prioritizes technical skills (e.g., measurement, costing, contract interpretation) at the expense of strategic and managerial training [10]. Instruction tends to focus on the “how” of quantity surveying without delving into the “why” of business decisions. Additionally, teaching methods often fail to incorporate realistic, project-based scenarios that simulate hands-on managerial challenges. Consequently, while graduates enter the industry with solid technical foundations, they frequently lack the strategic acumen for cost planning, financial management, and understanding broader client objectives—skills that industry experts acquire through direct experience.

The discrepancy in “personal image” skills can be attributed to an institutional emphasis on hard skills over professional socialization. The Chinese higher vocational curriculum prioritizes technical and academic mastery, offering limited formal instruction in soft skills like self-presentation. Consequently, graduates often lack exposure to practical exercises—such as presentations or networking simulations—that are critical for developing professional confidence and tacit knowledge. This deficit can impede their ability to assert ideas, cultivate professional networks, and decode unspoken corporate communication norms, highlighting a disjointed transition from student to professional roles.

The gap in “innovative insight” represents a systemic issue, rooted in the pedagogical foundations of Chinese higher vocational education. The quantity surveying curriculum’s deep focus on standardized processes (e.g., BoQ, Quota) and technical compliance inevitably marginalizes creative problem-solving. This tendency is reinforced by a pedagogical model favoring the passive absorption of “correct answers” rather than the active generation of multiple solutions. Furthermore, a national testing culture that rewards conformity further solidifies this approach. Ultimately, this gap is not a minor skill deficiency but the direct result of an educational environment that does not yet valorize the cognitive flexibility and intellectual risk-taking essential for modern industry challenges.

As a consequence, Table 8 summarizes the findings of the earlier hypotheses and shows that H1,H2, and H4 are accepted, while H3 rejected.

**Table 8:** Hypothesis results.

Hypothesis		Result
H1	A gap exists in the knowledge domain, wherein the industry’s expectation for technical and regulatory knowledge surpasses the perceived competency of HVC graduates.	Accept
H2	A gap exists in the skills domain, specifically in applied technical skills, where industry requirements are predicted to exceed the proficiency level of recent graduates.	Accept
H3	There will be no gap in the ethics domain, as ethical principles are a foundational and consistently emphasized component of both HVC curricula and professional practice.	Reject
H4	A gap exists in the personality domain, reflecting a systemic underdevelopment of these attributes within the current practice-oriented HVC training modell.	Accept

The study emphasizes the value of educational initiatives that emphasize personality development and management. Through multimodal educational programs that cover management and personality attributes including creative insight and personal image, it places a strong emphasis on improving QS staff competencies. It also emphasizes how crucial it is for industry and academia to work together more [19]. These initiatives, which are crucial for developing effective management, a positive self-image, and creative insight, may involve hands-on training, construction projects, and other activities [36]. More and more studies supports individualised and customised learning strategies that take into account each person’s unique requirements and traits [9]. By modifying the content, structure, and presentation for every student, personalized learning seeks to adapt the educational process to each individual’s particular needs within a group [19].

The identified gaps in business administration, personal image, and innovative insight indicate specific areas for enhancing the practice-oriented HVC model. Our findings yield three targeted implications: (a) The pronounced business administration gap underscores that technical prowess must be complemented by strategic understanding. Therefore, curricula should integrate dedicated modules on construction business management and financial strategy, using real-world case studies to connect operational tasks with strategic objectives. (b) The gaps in personal image and innovative insight call for active learning approaches. Simulation-based exercises (e.g., client negotiations) and open-ended project-based learning are recommended to concurrently foster professional self-presentation and the creative problem-solving skills demanded by the industry. (c) The persistence of these gaps suggests a need for stronger academia-industry links. Establishing structured feedback mechanisms, such as industry advisory panels with curriculum input, can help dynamically align educational outcomes with evolving professional competencies.

## Conclusion

This study set out to precisely measure the competency gaps between industry expectations and the competencies of Quantity Surveying (QS) graduates from Chinese HVCs. Employing an innovative high-dimensional measurement error model (the MULFR model), our analysis reveals a distinct misalignment pattern. The most pronounced gap was identified within the “Personality” domain, with the specific competencies of business administration, personal image, and innovative insight emerging as the most critical areas for intervention. In contrast, gaps in foundational Knowledge, Skills, and Ethics were comparatively minimal.

These findings carry significant implications for both educational practice and industry. They underscore a systemic shortfall in China’s practice-oriented HVC system: while it successfully produces graduates with solid technical foundations, it falls short in cultivating the integrated management acumen and professional self-presentation skills demanded by a modernizing construction industry. This suggests that curriculum reform must look beyond technical prowess and explicitly integrate learning objectives focused on strategic thinking, personal branding, and innovative problem-solving. The study demonstrates that competency is multidimensional, and effective bridging efforts must target the specific dimensions where gaps are widest.

Several limitations of this study pave the way for future research. First, Ng et al. contend that the MULFR model’s premise that competency domains and indicators are independent may not be accurate, which might result in problems with multicollinearity [37]. The reference values of $R_{p}^{\text{2}}$ and the related competence gap $\left(\text{1}-R_{p}^{\text{2}}\right)$ differ between sources and fields of study, such as Sawilowsky and Cohen [38]. As a result, the method employed in this study to determine the amount of skill gaps is new and could need to be improved. Second, the use of a purposive sampling method, while strategic for capturing a wide spectrum of graduate experiences, means that the sample is not statistically representative of all Chinese quantity surveying HVC graduates. Consequently, the quantitative estimates of the competency gaps identified may not be directly generalizable to the entire population in a statistical sense. The findings are more indicative of the nature and types of gaps that can exist, rather than their precise prevalence nationwide. Therefore, the generalizability of these findings is primarily analytical or theoretical rather than statistical. The results provide a valuable conceptual framework and highlight critical areas of concern—such as business administration, personal image, and innovative insight—that are likely relevant in similar educational and industrial contexts within China. However, the extent of these gaps might vary in different regional economies or specific industry sectors. Future research could employ probability sampling techniques across multiple provinces to validate and quantify these competency gaps on a broader scale, further enhancing the external validity of the findings. Third, a limitation stems from its reliance on self-reported data collected via a single questionnaire at one point in time, which introduces the risks of common method variance (CMV) and various response biases, such as social desirability bias and recall bias. For instance, graduates might overestimate their competencies to present a favorable image, while industry professionals might base their expectations on idealized standards rather than daily realities. CMV could artificially inflate the observed relationships between variables. Therefore, future research should seek to triangulate these findings by employing multi-source and multi-method approaches. For example, objective assessments of graduate competencies (e.g., analysis of work portfolios or supervisor ratings) could be combined with the industry expectations survey. Longitudinal designs tracking graduates over time would also help circumvent the limitations of cross-sectional data and provide a more dynamic understanding of how competency gaps evolve.

Based on these limitations and our findings, we propose three clear directions for future work: Intervention Studies: To develop and test specific pedagogical interventions (e.g., case-based learning, simulated business projects) aimed at effectively cultivating personality-related competencies like innovative insight and business administration. Contextual Inquiry: To investigate how major disruptive events, such as the COVID-19 pandemic, have reshaped competency requirements and gaps in the QS profession. Comparative Analysis: To conduct cross-cultural comparative studies, contrasting these findings with data from other countries to disentangle universal trends from context-specific challenges in QS education.

In conclusion, by moving beyond a generic skills gap analysis to a multidimensional diagnostic, this study provides a precise roadmap for aligning HVC education with industry needs, ultimately contributing to the enhanced employability of graduates and the competitiveness of China’s construction sector.
